# Graded Electrolyte Disturbances Across the Spectrum of Autonomous Cortisol Secretion in Patients with Adrenal Incidentaloma: A Retrospective Cohort Study

**DOI:** 10.3390/jcm15145442

**Published:** 2026-07-11

**Authors:** Yanan Li, Xiang Yao, Xin Zhao, Yushi Zhang

**Affiliations:** 1 Department of Urology, Peking Union Medical College Hospital, Chinese Academy of Medical Science & Peking Union Medical College, Beijing 100730, China; liyanan35@pumch.cn; 2 Chinese Academy of Medical Sciences & Peking Union Medical College, Beijing 100730, China; b2024012008@student.pumc.edu.cn

**Keywords:** adrenal incidentaloma, autonomous cortisol secretion, mild autonomous cortisol secretion, Cushing’s syndrome, electrolyte homeostasis, renal function, eGFR

## Abstract

**Background/Objectives:** Autonomous cortisol secretion (ACS), including mild autonomous cortisol secretion (MACS), nonfunctioning adrenal tumors (NFAT), and overt Cushing’s syndrome (CS), is common in patients with adrenal incidentaloma (AI). Although ACS has been linked to adverse cardiometabolic outcomes, its association with renal function and electrolyte homeostasis has not been well characterized. To examine the associations of different degrees of ACS with renal function and serum electrolyte profiles in patients with AI. **Methods:** This retrospective single-center study included 575 adult patients with AI who underwent an overnight low-dose dexamethasone suppression test (LDDST). Patients were classified as having NFAT (*n* = 30), MACS (*n* = 236), or overt CS (*n* = 309) according to biochemical findings and clinical presentation. Clinical characteristics, hormonal parameters, estimated glomerular filtration rate (eGFR), and serum electrolyte levels were collected. Multivariable regression analyses were performed after adjustment for age, sex, tumor size, body mass index, hypertension, diabetes mellitus, smoking status, and alcohol consumption. **Results:** Compared with the NFAT and MACS groups, patients with overt CS were younger and predominantly female (84.5%), more frequently hypertensive, and had the highest serum and urinary free cortisol, the lowest ACTH, and the most pronounced electrolyte disturbances, with higher serum sodium and lower serum potassium (all *p* < 0.001). A pattern of electrolyte alterations was observed across the spectrum of cortisol autonomy (CS ≈ MACS > NFAT). Compared with NFAT, cortisol-secreting tumors were associated with higher serum sodium levels and lower serum potassium and calcium levels (*p* < 0.001). These associations remained significant after multivariable adjustment (*p* < 0.001). By contrast, although median eGFR differed among groups and tended to be higher in cortisol-secreting tumors, ACS was not significantly associated with clinically overt renal impairment based on categorical eGFR analysis. **Conclusions:** In patients with AI, increasing degrees of ACS are associated with alterations in electrolyte homeostasis, particularly involving sodium, potassium, and calcium. In contrast, no clear association was identified between ACS and clinically overt renal impairment in this cross-sectional cohort. Routine monitoring of electrolyte balance may be warranted in patients with cortisol-secreting adrenal tumors.

## 1. Introduction

Adrenal incidentalomas (AIs) are adrenal masses incidentally detected on imaging studies in the absence of clinical suspicion for adrenal disease. In most cases, these lesions are nonfunctioning adrenocortical adenomas; however, approximately 40% of patients with AIs exhibit varying degrees of autonomous cortisol hypersecretion [1]. The term “mild autonomous cortisol secretion” (MACS) was coined to describe the state of subclinical mild cortisol excess [2]. In contrast to overt Cushing’s syndrome, MACS lacks classic clinical manifestations. Notably, MACS is associated with an increased risk of cardiovascular and metabolic abnormalities, as well as bone metabolic disorders including osteoporosis [3,4].

**Figure 1:**
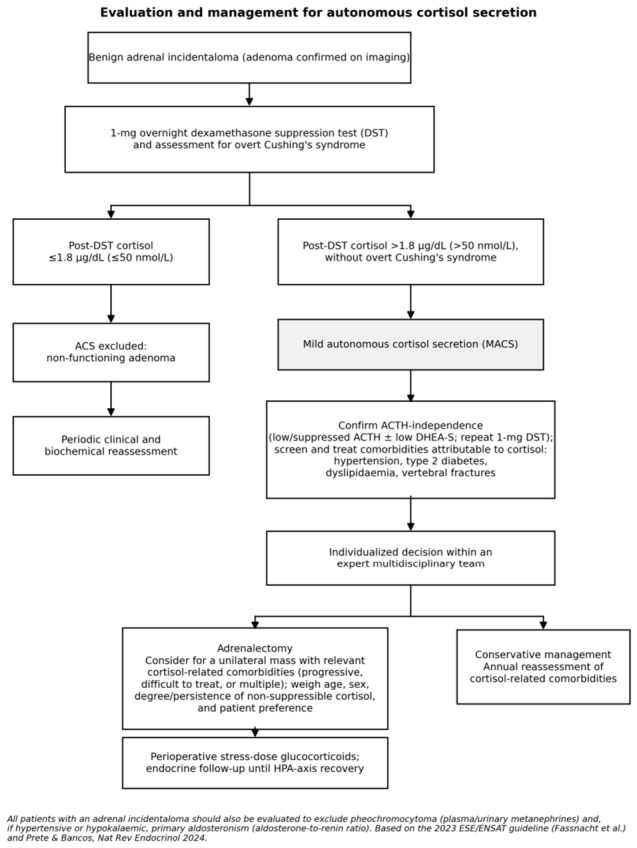
Evaluation and management for autonomous cortisol secretion. After a benign adenoma is confirmed, all patients undergo a 1 mg overnight dexamethasone suppression test (DST). A post-DST serum cortisol ≤1.8 µg/dL (≤50 nmol/L) excludes autonomous cortisol secretion, whereas a value >1.8 µg/dL (>50 nmol/L) without overt Cushingoid features defines mild autonomous cortisol secretion (MACS). In MACS, ACTH-independence is confirmed and cortisol-related comorbidities are screened and treated; management (adrenalectomy versus conservative follow-up) is then individualized within a multidisciplinary team. Adapted from the 2023 ESE/ENSAT guideline [2] and Prete & Bancos [10]. DST, dexamethasone suppression test; MACS, mild autonomous cortisol secretion; ACS, autonomous cortisol secretion.

AI have also become increasingly common with the widespread use of cross-sectional imaging. In a large unselected screening study of more than 25,000 adults, the prevalence of adrenal tumors was 1.4%, increasing with age from 0.2% in participants aged 18 to 25 years to 3.2% in those older than 65 years, with the great majority being adrenocortical adenomas [5]. Despite this high and rising prevalence, many such lesions remain under-recognized in routine practice, and a recent tertiary-center study reported a high frequency of adrenal incidentalomas accompanied by a low rate of appropriate endocrine referral [6]. A substantial proportion of these tumors are hormonally active, most often through MACS, with overt adrenal Cushing’s syndrome representing the severe end of this spectrum. Nationwide cohort data have shown that adrenal Cushing’s syndrome, although uncommon, is a major subtype of Cushing’s syndrome and carries a considerable burden of long-term comorbidity [7]. Together, these observations highlight the clinical importance of characterizing the metabolic consequences of autonomous cortisol secretion across its full spectrum, from non-functioning adrenal tumors through MACS to overt Cushing’s syndrome. The current clinical work-up and management of an autonomous cortisol-secreting adrenal incidentaloma are summarized (Figure 1).

While previous studies have investigated comprehensive comparisons of demographic, pathological and biochemical features across adrenal incidentalomas, it has also remained limited regarding the associations with renal function and electrolyte homeostasis. Existing evidence highlights that adrenal tumors, even non-functional ones, are linked to systemic inflammation and metabolic dysregulation [8]. Yet, few studies have systematically evaluated electrolyte abnormalities and renal function across NFAT, MACS, and CS, despite the recognized risk of renal impairment in hypercortisolemic states. The only paper discussing the effect of Cushing Syndrome on kidney function was not even tested on humans but on dogs [9].

This study aims to compare the renal functional risks and electrolyte abnormalities between MACS or Cushing’s syndrome and NFAT, and highlight the need for routine monitoring of renal function and electrolytes in AI patients, providing evidence-based guidance for clinical decision-making.

## 2. Methods

### 2.1. Data Collection

Our study was approved by the Institutional Review Board of Peking Union Medical College Hospital (Approval No.: I-24PJ1048). Written or verbal informed consent was obtained from all enrolled patients. All information was anonymized.

**Figure 2:**
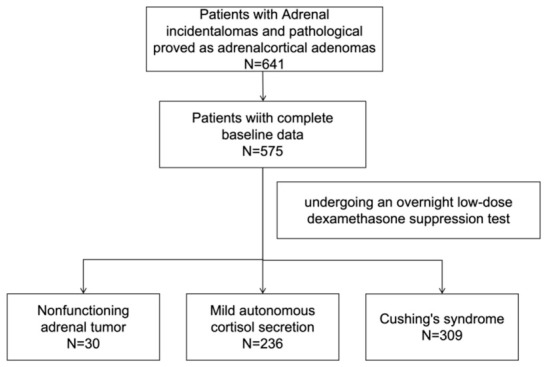
Flow diagram of patient selection. Of the 641 patients with adrenal incidentalomas pathologically confirmed as adrenocortical adenomas, 66 were excluded owing to missing key clinical data and pathologically proven adrenal medullary tumors, adrenocortical carcinoma, adrenocortical hyperplasia, or other unclassified adrenal tumors, leaving 575 patients who underwent the overnight 1 mg dexamethasone suppression test and were classified as non-functioning adrenal tumor (*n* = 30), mild autonomous cortisol secretion (*n* = 236), or Cushing’s syndrome (*n* = 309).

Patients with adrenal incidentalomas (AIs) who were hospitalized at Peking Union Medical College Hospital over the past decade were retrospectively enrolled and evaluated. The inclusion criteria were as follows: (i) age ≥ 18 years; (ii) completion of an overnight low-dose dexamethasone suppression test (LDDST); and (iii) availability of complete clinical data, including hormonal assays, imaging findings, and biochemical indicators. The exclusion criteria were as follows: (i) pathologically proven adrenal medullary tumors, adrenocortical carcinoma, adrenocortical hyperplasia, or other unclassified adrenal tumors; and (ii) missing key clinical data (e.g., hormonal assay results, renal function parameters, and electrolyte profiles). All patients were diagnosed as having overt Cushing’s syndrome, mild autonomous cortisol secretion (MACS), or nonfunctioning adrenal tumor (NFAT) based on the results of the overnight LDDST and clinical manifestations (Figure 2).

All patients underwent standardized clinical assessments, which included the following: (i) medical history collection (chief complaints, comorbidities [e.g., diabetes mellitus, hypertension (HT), smoking history, and alcohol consumption history]); (ii) physical examination; (iii) laboratory tests; and (iv) imaging examinations. Clinical data were extracted by two independent researchers and cross-checked to ensure data accuracy.

8 a.m. serum cortisol (F8am), 24-h urinary free cortisol (24hUFC), and adrenocorticotropic hormone (ACTH) levels were identified as the primary outcome measures in this study. The reference ranges for F8am and ACTH were 6.3–22.3 μg/dL and 7.2–63.3 pg/mL, respectively. Relevant clinical manifestations encompassed decreased libido, moon facies, menstrual irregularities, hirsutism, cutaneous bruising, fatigue, depressive symptoms, purple striae, and proximal myopathy. According to the clinical practice guidelines formulated by the European Society of Endocrinology and the European Network for the Study of Adrenal Tumors (ESE-ENSAT), the 1 mg dexamethasone suppression test (1 mg-DST) is the recommended diagnostic tool for mild autonomous cortisol secretion (MACS). Overt Cushing’s syndrome was diagnosed in patients with adrenal tumors and corresponding clinical manifestations who failed to achieve adequate serum cortisol suppression following an overnight low-dose dexamethasone suppression test (LDDST). MACS was defined as the absence of classic Cushing’s syndrome clinical features in patients with at least one adrenocortical tumor, accompanied by insufficient serum cortisol suppression after an overnight LDDST (cortisol > 50 nmol/L (>1.8 µg/dL)). Nonfunctioning adrenal tumors (NFAT) were characterized by successful serum cortisol suppression (cortisol < 50 nmol/L) following an overnight 1 mg-DST. To verify the accuracy of this classification, 24-h urinary free cortisol (UFC) was assessed in the MACS and CS groups; an elevated UFC was defined as >60 µg/24 h in men and >45 µg/24 h in women [11,12].

Diabetes mellitus was diagnosed according to the 2016 American Diabetes Association Standards of Medical Care in Diabetes [13]. HT was diagnosed according to the American College of Cardiology guidelines [14]. Body mass index (BMI) was calculated as weight (kg)/height^2^ (m^2^). Serum electrolytes included serum potassium(K) (3.5–5.5 mmol/L), sodium(Na) (135–145 mmol/L), chloride(Cl) (96–111 mmol/L), calcium(Ca) (2.11–2.52 mmol/L), and phosphorus(P) (0.81–1.45 mmol/L); reference ranges provided respectively. Serum calcium values were corrected for serum albumin.

Kidney function was assessed by estimated glomerular filtration rate (eGFR) and serum electrolytes (potassium, sodium, chloride, calcium, phosphorus). eGFR was computed using the Chronic Kidney Disease Epidemiology Collaboration (CKD-EPI) equation [15], incorporating serum creatinine (μmol/L), age, and gender. eGFR < 90mL/min/1.73 m^2^ was considered as renal impairment.

Covariates included gender, age, maximum tumor diameter, hypertension, diabetes, smoking, alcohol consumption, and BMI.

### 2.2. Statistical Analysis

For descriptive statistical analyses, continuous variables (e.g., age, BMI, maximum tumor diameter, hormonal levels, eGFR and electrolyte levels) were reported as mean ± standard deviation (SD) or median (interquartile range, IQR) as appropriate. Intergroup comparisons were conducted with specific statistical methods based on group numbers: independent samples t-tests or nonparametric tests (e.g., Mann–Whitney U test) were applied for two-group comparisons, while one-way analysis of variance (ANOVA) was used to compare variables across three groups. For categorical variables (e.g., sex, hypertension, diabetes, eGFR grading), data were summarized as frequencies (percentages), and group differences were examined using chi-square tests or Fisher’s exact tests where applicable. Odds ratios (ORs) with 95% confidence intervals (CIs) were computed to quantify the differences among the Cushing’s syndrome, MACS and NFAT groups. Univariate analyses were performed to compare the baseline clinical characteristics of the three study groups. A multivariate analysis model was constructed, with gender, age, maximum tumor diameter, hypertension, diabetes, smoking status, alcohol consumption, and BMI included as covariates. Multiple imputation was adopted to impute missing data points, aiming to mitigate potential selection bias in the study. All statistical analyses were implemented using R software (the latest stable version available at www.R-project.org) and Free Statistics 1.9 software. Statistical significance was determined as a two-tailed *p*-value < 0.05 for all tests. To distinguish statistical significance from clinical relevance, effect sizes were additionally calculated for the pairwise electrolyte comparisons as r = Z/√(n1 + n2), with r values of approximately 0.1, 0.3, and 0.5 regarded as small, medium, and large, respectively.

## 3. Results

A total of 575 patients were enrolled, including 30 in the NFAT group, 236 in the MACS group, and 309 in the Cushing group. The demographic and clinical characteristics of the study population are summarized in Table 1. Cushing group had the highest female proportion (84.5%) and youngest age (all *p* < 0.001), plus higher hypertension prevalence (*p* = 0.034). Biochemical differences were prominent in the Cushing group: markedly higher 8am cortisol and UFC, lowest ACTH (all *p* < 0.001); higher Na, lower K and phosphate (Na *p* < 0.001, K *p* = 0.003, phosphate *p* = 0.002) vs. other groups.

To explore association between cortisol secretion and renal function, we analyzed eGFR distribution in 575 patients (Table 2 and Table 3). Most had eGFR > 90 mL/min/1.73 m^2^; chi-squared test showed no significant intergroup eGFR difference (*p* = 0.128), indicating cortisol secretion does not affect renal function clinically.

Most electrolytes differed significantly across NFAT, MACS and Cushing groups (Table 4). MACS/Cushing had positive associations with Na (OR = 1.16/1.64, all *p* < 0.001) and Cl (OR = 1.25/1.26, all *p* < 0.011), inverse with K (OR = 0.17/0.14, *p* ≤ 0.001). Cushing had a strong negative association with Ca (OR = 0, *p* < 0.001); *p* had no significant associations (all *p* > 0.289).

Multivariate logistic regression (MACS as control, confounders adjusted) revealed significant electrolyte differences between Cushing syndrome and MACS (Table 5): Cushing had higher Na (*p* < 0.001) with lower sodium-water retention and K (*p* < 0.001). It also had markedly lower Ca (*p* < 0.001). Electrolyte (Na/K/Cl/Ca) associations were hierarchical across NFAT, MACS and Cushing, persisting after adjustment; electrolyte imbalance severity was ranked Cushing ≈ MACS > NFAT.

To further clarify the distinction between statistical significance and effect size for sodium, potassium, and calcium, pairwise comparisons with their corresponding effect sizes are presented in Supplementary Table S1. Both MACS and CS showed distinct electrolyte deviations from NFAT (*p* < 0.05 with r > 0.3), whereas the differences between MACS and CS, although statistically significant, were of smaller or even negligible magnitude (r ranging from 0.098 to 0.214).

## 4. Discussion

Overt Cushing’s syndrome causes sodium–water retention in clinical practice. However, there is currently a lack of systematic analysis regarding the impact of different degrees of cortisol secretion on renal function and electrolytes. This study is the first cross-sectional study to systematically analyze the renal and electrocytic features of patients with NFAT, mild autonomous cortisol secretion (MACS), and overt Cushing’s syndrome. A total of 575 patients were enrolled in this study, including 30 cases of NFAT, 236 of MACS, and 309 of overt Cushing’s syndrome. Adrenal tumors with cortisol hypersecretion were found to be associated with significant elevations in serum sodium levels as well as a reduction in serum potassium and calcium levels. Overt Cushing’s syndrome was accompanied by the most severe electrolyte imbalance, almost followed by MACS, and both were more pronounced than those in NFAT patients. The underlying mechanisms are primarily linked to the non-specific activation of mineralocorticoid receptors (MR) by cortisol [16]. Under normal physiological conditions, this non-specific activation is constrained by 11β-hydroxysteroid dehydrogenase type 2 (11β-HSD2). In pathological states (e.g., chronic stress, Cushing’s syndrome), the non-specific MR activation by cortisol may be exacerbated, thereby promoting sodium-water retention, hypokalemia, and even metabolic alkalosis [17,18]. No studies have summarized electrolyte abnormalities (e.g., hypernatremia, hypokalemia) in autonomous cortisol secretion (ACS). Our study quantitatively demonstrated that adrenal tumors with autonomous cortisol secretion are closely associated with electrolyte abnormality, including elevated serum sodium, reduced serum potassium and calcium levels.

The prevalence of renal impairment in the cohort was 13.2%; despite eGFR being slightly higher in Cushing and MACS, cortisol secretion had no clinically meaningful impact on renal function in this cohort. Only one retrospective study has reported that MACS combined with primary aldosteronism (PA) increases the risk of renal complications, mainly manifested as elevated eGFR and proteinuria [19]. In our study, a trend toward elevated eGFR was observed in patients with autonomous cortisol secretion, without statistical significance. It may be attributed to the small sample size of the NFAT and the absence of PA comorbidity in the study population, indicating that the direct renal damage induced by autonomous cortisol secretion is milder compared with that by primary aldosteronism.

In the present cohort, serum calcium decreased progressively across the spectrum of cortisol autonomy, being lowest in overt Cushing’s syndrome. Rather than being a direct consequence of osteoporosis, this reduction may be partly explained by the effects of glucocorticoid excess on whole-body calcium handling: cortisol decreases intestinal calcium absorption while simultaneously increasing urinary calcium loss through impaired renal tubular reabsorption, and the resulting negative calcium balance lowers circulating calcium levels [20]. The greater degree of cortisol autonomy in overt Cushing’s syndrome would be expected to amplify both of these effects, which is consistent with the graded pattern we observed, in which more pronounced cortisol excess was accompanied by greater reductions in serum calcium [21].

Among the few studies, we are the first cross-sectional and clinical study to systematically elucidate the impacts of autonomous cortisol secretion on renal function and electrolyte homeostasis in a relatively large cohort with clear stratification by the degree of cortisol secretion, emphasizing the need for targeted electrolyte correction and hypertension management in AI patients, with Cushing patients requiring closer monitoring of electrolyte disturbances and sodium-water retention. We combined univariate and multivariate logistic regression to confirm independent associations.

This study has several limitations. First, the sample size was relatively small and the study was single-centered, which may have introduced selection bias. Additionally, the small sample size of the NFAT group limited the statistical power for subgroup analyses. Furthermore, the lack of long-term follow-up data precluded an investigation into the dynamic changes in electrolytes and renal function after clinical intervention. Future research will include multi-centered, large-sample studies with extended long-term follow-up. In addition, although there were statistically significant differences between the groups, the median differences between the groups were relatively small and all fell within the normal reference range, indicating a limited clinical discriminatory ability. We also plan to explore the underlying molecular mechanisms responsible for the graded electrolyte disturbances observed across different subtypes of adrenal tumors with cortisol hypersecretion. The last point is because all included patients were inpatients, whereas most NFAT patients were managed and followed as outpatients; the proportion of MACS and CS relative to NFAT in this cohort is higher than in unselected populations, which may have introduced bias.

## 5. Conclusions

Our study results reveal that different degrees of autonomous cortisol secretion are associated with hierarchical electrolyte imbalance (Cushing > MACS > NFAT), which is an independent characteristic and has important therapeutic and prognostic implications for clinical practice, Elevated autonomous cortisol secretion was not found to be significantly associated with clinically renal impairment, but we found a increasing trend between cortisol-secreting-tumor and NFAT, suggesting that cortisol may exert a mild effect on renal function, although far lesser than primary aldosteronism. We recommend that patients with autonomous cortisol secretion should monitor electrolyte disturbances and sodium-water retention. Future multicenter, large-sample cohort studies are needed to further validate our findings.

## Figures and Tables

**Table 1:** Baseline demographic and clinical characteristics of the study population.

Variables	Total (*n* = 575)	NFAT (*n* = 30)	MACS (*n* = 236)	Cushing (*n* = 309)	*p*
gender, *n* (%)					<0.001
male	134 (23.3)	13 (43.3)	73 (30.9)	48 (15.5)	
female	441 (76.7)	17 (56.7)	163 (69.1)	261 (84.5)	
position, *n* (%)					0.358
single	442 (76.9)	26 (86.7)	183 (77.5)	233 (75.4)	
bilateral	133 (23.1)	4 (13.3)	53 (22.5)	76 (24.6)	
hypertension, *n* (%)					0.034
absence	189 (32.9)	15 (50)	84 (35.6)	90 (29.1)	
presence	386 (67.1)	15 (50)	152 (64.4)	219 (70.9)	
diabetes, *n* (%)					0.108
absence	443 (77.0)	27 (90)	186 (78.8)	230 (74.4)	
presence	132 (23.0)	3 (10)	50 (21.2)	79 (25.6)	
smoking, *n* (%)					<0.001
absence	488 (84.9)	22 (73.3)	185 (78.4)	281 (90.9)	
presence	87 (15.1)	8 (26.7)	51 (21.6)	28 (9.1)	
alcohol consumption, *n* (%)					0.915
absence	486 (84.5)	25 (83.3)	201 (85.2)	260 (84.1)	
presence	89 (15.5)	5 (16.7)	35 (14.8)	49 (15.9)	
age, Median (IQR)	45.0 (35.0, 55.0)	49.5 (44.0, 54.0)	50.0 (42.0, 59.0)	39.0 (30.0, 51.0)	<0.001
maximum tumor diameter (cm), Median (IQR)	2.7 (2.1, 3.1)	2.2 (1.8, 2.5)	2.6 (2.0, 3.1)	2.8 (2.4, 3.1)	<0.001
BMI, Median (IQR)	25.0 (22.7, 27.7)	24.3 (22.5, 27.6)	25.6 (23.3, 27.9)	24.7 (22.5, 27.2)	0.073
F8am, Median (IQR)	21.1 (14.2, 26.5)	12.4 (10.4, 14.5)	15.3 (11.1, 21.2)	24.6 (20.7, 29.4)	<0.001
UFC, Median (IQR)	224.7 (94.2, 506.0)	78.0 (51.7, 113.0)	102.0 (58.5, 169.0)	451.0 (258.7, 684.1)	<0.001
ACTH, Median (IQR)	5.0 (5.0, 8.1)	11.9 (5.0, 19.5)	5.8 (5.0, 9.6)	5.0 (5.0, 5.0)	<0.001
Na, Median (IQR)	141.0 (140.0, 142.0)	140.0 (139.0, 141.0)	140.0 (139.0, 142.0)	141.0 (140.0, 143.0)	<0.001
K, Median (IQR)	4.0 (3.7, 4.2)	4.1 (3.9, 4.3)	4.0 (3.8, 4.2)	3.9 (3.6, 4.3)	0.003
Cl, Median (IQR)	105.0 (104.0, 107.0)	105.0 (103.2, 106.0)	106.0 (104.0, 107.0)	105.0 (103.0, 107.0)	0.137
Ca, Mean ± SD	2.3 ± 0.1	2.3 ± 0.1	2.3 ± 0.1	2.3 ± 0.1	<0.001
P, Median (IQR)	1.2 (1.0, 1.3)	1.2 (1.0, 1.3)	1.2 (1.1, 1.3)	1.1 (1.0, 1.3)	0.002

Abbreviations: NFAT(non-functional adrenal tumor), MACS (Mild Autonomous Cortisol Secretion), BMI (body mass index), UFC (urinary free cortisol), F8am (serum cortisol at 8am), ACTH (adrenocorticotropic hormone), Na (sodium), K (potassium), Cl (chloride), Ca (calcium), P(phosphorus); *p*-values indicate statistical significance of intergroup differences (calculated using chi-square tests for categorical variables and one-way analysis of variance for continuous variables). *p* < 0.05, two-sided.

**Table 2:** Distribution of estimated glomerular filtration rate (eGFR) categories in the total cohort and subgroup analyses.

eGFR (mL/min/1.73 m^2^)	Total (*n* = 575)	NFAT (*n* = 30)	MACS (*n* = 236)	Cushing (*n* = 309)	*p*
categories, *n* (%)					0.128
<60	12 (2.1)	0 (0)	1 (0.4)	11 (3.6)	
60–90	64 (11.1)	2 (6.7)	28 (11.9)	34 (11)	
>90	499 (86.8)	28 (93.3)	207 (87.7)	264 (85.4)	
Median (IQR)	106.4 (95.8, 117.0)	109.2 (98.8, 112.3)	102.4 (93.4, 111.7)	109.8 (97.7, 120.7)	<0.001

Abbreviations: NFAT (non-functional adrenal tumor), MACS (Mild Autonomous Cortisol Secretion), eGFR (estimated glomerular filtration rate); *p*-values indicate statistical significance of intergroup differences (calculated using chi-square tests for categorical variables and one-way analysis of variance for continuous variables). *p* < 0.05, two-sided.

**Table 3:** Distribution of estimated glomerular filtration rate (eGFR) categories in the subgroup analyses.

Variable	Total, *n*	NFAT, *n* (%)	MACS, *n* (%)	Cushing, *n* (%)	MACS vs. NFAT, OR (95% CI)	*p* Value	Cushing vs. NFAT, OR (95% CI)	*p* Value
eGFR	575	30 (5.2)	236 (41)	309 (53.7)	0.98 (0.95~1.02)	0.315	0.98 (0.94~1.01)	0.169

Abbreviations: NFAT (non-functional adrenal tumor), MACS (Mild Autonomous Cortisol Secretion), eGFR (estimated glomerular filtration rate); Multivariate logistic regression analysis was used to calculate odds ratios (OR) and 95% confidence intervals (95% CI). A two-tailed *p*-value < 0.05 was considered statistically significant.

**Table 4:** Comparison of electrolyte abnormalities among different study groups.

Variable	Total, *n*	NFAT, *n* (%)	MACS, *n* (%)	Cushing, *n* (%)	MACS vs. NFAT, OR (95% CI)	*p* Value	Cushing vs. NFAT, OR (95% CI)	*p* Value
Na	575	30 (5.2)	236 (41)	309 (53.7)	1.16 (0.92~1.46)	0.202	1.64 (1.32~2.03)	<0.001
K	575	30 (5.2)	236 (41)	309 (53.7)	0.17 (0.05~0.57)	0.004	0.14 (0.04~0.46)	0.001
Cl	575	30 (5.2)	236 (41)	309 (53.7)	1.25 (1.02~1.54)	0.03	1.26 (1.05~1.5)	0.011
Ca	575	30 (5.2)	236 (41)	309 (53.7)	0.01 (0~0.87)	0.043	0 (0~0.01)	<0.001

Abbreviations: NFAT (non-functional adrenal tumor), MACS (Mild Autonomous Cortisol Secretion), Na (sodium), K (potassium), Cl (chloride), Ca (calcium), P (phosphorus); Multivariate logistic regression analysis was used to calculate odds ratios (OR) and 95% confidence intervals (95% CI). A two-tailed *p*-value < 0.05 was considered statistically significant.

**Table 5:** The comparison of electrolyte abnormalities between Cushing and MACS Syndrome.

Variable	Total, *n*	Events, *n* (%)	Crude OR (95% CI)	Crude P	Adjusted OR (95% CI)	Adjusted P
Na	545	309 (53.7)	1.2 (1.11~1.3)	<0.001	1.47 (1.33~1.64)	<0.001
K	545	309 (53.7)	0.52 (0.34~0.78)	0.002	0.37 (0.22~0.62)	<0.001
Cl	545	309 (53.7)	0.97 (0.91~1.04)	0.442	1.09 (1.01~1.18)	0.031
Ca	545	309 (53.7)	0.04 (0.01~0.19)	<0.001	0.01 (0~0.08)	<0.001

Abbreviations: MACS (Mild Autonomous Cortisol Secretion), Na (sodium), K (potassium), Cl (chloride), Ca (calcium), P (phosphorus); Multivariate logistic regression analysis was used to calculate odds ratios (OR) and 95% confidence intervals (95% CI). A two-tailed *p*-value < 0.05 was considered statistically significant.

## Data Availability

The raw data supporting the conclusions of this article will be made available by the corresponding author on request.

## Supplementary Materials

The following supporting information can be downloaded at: https://www.mdpi.com/article/10.3390/jcm15145442/s1, Table S1: Pairwise comparisons of serum sodium, potassium, and calcium among the NFAT, MACS, and Cushing's syndrome groups, showing both statistical significance and effect size (Mann–Whitney U test).
