# Supplementary material for: Graded Electrolyte Disturbances Across the Spectrum of Autonomous Cortisol Secretion in Patients with Adrenal Incidentaloma: A Retrospective Cohort Study
Source: J Clin Med. 2026 Jul 11;15(14):5442. doi: 10.3390/jcm15145442 (PMC13412405; doi:10.3390/jcm15145442)
Supplement: Supplementary file 1 [file jcm-15-05442-s001.zip › jcm-4372803-supplementary.pdf]

Supplement Table S1. Pairwise comparisons of serum sodium, potassium, and calcium among the NFAT, MACS, and Cushing's syndrome groups, showing both statistical significance and effect size (Mann–Whitney U test).

Electrolyte	Comparison	n1/n2	Rank1/Rank2	Z Value	P Value	Effect Size (r)
**Na**	NFAT vs MACS	30/236	58.37/143.05	-5.738	<0.001	0.352
	NFAT vs Cushing	30/309	56.25/181.04	-6.704	<0.001	0.364
	MACS vs Cushing	236/309	234.75/302.21	-5.001	<0.001	0.214
**K**	NFAT vs MACS	30/236	205.63/124.33	-5.475	<0.001	0.336
	NFAT vs Cushing	30/309	270.75/160.22	-5.910	<0.001	0.321
	MACS vs Cushing	236/309	290.57/259.58	-2.284	0.022	0.098
**Ca**	NFAT vs MACS	30/236	203.83/124.56	-5.319	<0.001	0.326
	NFAT vs Cushing	30/309	273.98/159.90	-6.089	<0.001	0.331
	MACS vs Cushing	236/309	303.46/249.74	-3.948	<0.001	0.169

The comparative analysis demonstrated that both MACS and Cushing exhibited distinct electrolyte deviations from NFAT patients, while the differences between MACS and Cushing remained relatively modest. Specifically, compared with the NFAT group, both MACS and Cushing groups showed significantly elevated serum sodium levels along with markedly depressed potassium and calcium levels ($P<0.05$ with $r > 0.3$). In contrast, the electrolyte variations between MACS and Cushing patients, though statistically significant, presented smaller or even negligible effect magnitudes (ranging from $r = 0.098$ to 0.214). P values are based on asymptotic two-tailed significance. The effect size is calculated as $r = |Z|/\sqrt{n1 + n2}$
